# Examining Whether AOSLO-Based Foveal Cone Metrics in Achromatopsia and Albinism Are Representative of Foveal Cone Structure

**DOI:** 10.1167/tvst.10.6.22

**Published:** 2021-05-17

**Authors:** Katie M. Litts, Erica N. Woertz, Niamh Wynne, Brian P. Brooks, Alicia Chacon, Thomas B. Connor, Deborah Costakos, Alina Dumitrescu, Arlene V. Drack, Gerald A. Fishman, William W. Hauswirth, Christine N. Kay, Byron L. Lam, Michel Michaelides, Mark E. Pennesi, Kimberly E. Stepien, Sasha Strul, C. Gail Summers, Joseph Carroll

**Affiliations:** 1 Department of Ophthalmology & Visual Sciences, Medical College of Wisconsin, Milwaukee, WI, USA; 2 Department of Cell Biology, Neurobiology and Anatomy, Medical College of Wisconsin, Milwaukee, WI, USA; 3 School of Medicine, Medical College of Wisconsin, Milwaukee, WI, USA; 4 National Eye Institute, Bethesda, MD, USA; 5 Department of Ophthalmology and Visual Sciences, University of Iowa Hospitals and Clinics, Iowa City, IA, USA; 6 Department of Ophthalmology and Visual Sciences, University of Illinois at Chicago, Chicago, IL, USA; 7 Department of Ophthalmology, University of Florida, Gainesville, FL, USA; 8 Vitreoretinal Associates, Gainesville, FL, USA; 9 Bascom Palmer Eye Institute, University of Miami, Miami, FL, USA; 10 UCL Institute of Ophthalmology, University College London, London, UK; 11 Moorfields Eye Hospital NHS Foundation Trust, London, UK; 12 Casey Eye Institute, Oregon Health & Science University, Portland, OR, USA; 13 Department of Ophthalmology and Visual Sciences, University of Wisconsin–Madison, Madison, WI, USA; 14 Department of Ophthalmology & Visual Neurosciences, University of Minnesota, Minneapolis, MN, USA

**Keywords:** adaptive optics scanning light ophthalmoscopy, achromatopsia, albinism, foveal cones

## Abstract

**Purpose:**

Adaptive optics scanning light ophthalmoscopy (AOSLO) imaging in patients with achromatopsia (ACHM) and albinism is not always successful. Here, we tested whether optical coherence tomography (OCT) measures of foveal structure differed between patients for whom AOSLO images were either quantifiable or unquantifiable.

**Methods:**

The study included 166 subjects (84 with ACHM; 82 with albinism) with previously acquired OCT scans, AOSLO images, and best-corrected visual acuity (BCVA, if available). Foveal OCT scans were assessed for outer retinal structure, outer nuclear layer thickness, and hypoplasia. AOSLO images were graded as quantifiable if a peak cone density could be measured and/or usable if the location of peak density could be identified and the parafoveal mosaic was quantifiable.

**Results:**

Forty-nine percent of subjects with ACHM and 57% of subjects with albinism had quantifiable AOSLO images. Older age and better BCVA were found in subjects with quantifiable AOSLO images for both ACHM (*P* = 0.0214 and *P* = 0.0276, respectively) and albinism (*P* = 0.0073 and *P* < 0.0004, respectively). There was a significant trend between ellipsoid zone appearance and ability to quantify AOSLO (*P* = 0.0028). In albinism, OCT metrics of cone structure did not differ between groups.

**Conclusions:**

Previously reported AOSLO-based cone density measures in ACHM may not necessarily reflect the degree of remnant cone structure in these patients.

**Translational Relevance:**

Until AOSLO is successful in all patients with ACHM and albinism, the possibility of the reported data from a particular cohort not being representative of the entire population remains an important issue to consider when interpreting results from AOSLO studies.

## Introduction

Adaptive optics scanning light ophthalmoscopy (AOSLO) allows for single-cell resolution of photoreceptors in health and disease, permitting non-invasive interrogation of pathology and elucidation of the clinical appearance of retinal disease. The normal photoreceptor mosaic has been thoroughly studied by AOSLO^1^^,^^2^ and shown to agree with observations from prior histology reports.^3^^,^^4^ As acquiring extensive histology from patients with inherited diseases affecting the retina is not practicable, AOSLO serves as the main tool to characterize photoreceptor pathology in these conditions in vivo. With numerous clinical trials planned or ongoing for many retinal diseases, photoreceptor-based biomarkers from AOSLO images may be useful in monitoring the safety and efficacy of therapeutic interventions aimed at restoring cone function. Despite the utility of AOSLO and advances in its application, it is not yet successful in everyone, even in some individuals without retinal disease, because of fatigue from the long imaging session, dry eyes, cataract, or poor optics of the eye.^5^ As such, data reported to date may provide at best an incomplete picture of the condition(s) of interest and may at worst be misleading.

One disease where AOSLO has been used extensively is achromatopsia (ACHM). Recent findings in the most common genetic subtypes of ACHM (*CNGA3* and *CNGB3*) demonstrate the presence of remnant cone structure, with peak cone density being highly variable among subjects,^6^^,^^7^ which could predict corresponding differences in therapeutic potential. Additionally, strong within-subject interocular symmetry has been demonstrated,^8^ and little or no change in peak cone density (over 2-year follow-up) has been observed in ACHM.^9^ The degree of remnant cone structure is even more variable among patients with ACHM associated with mutations in other genes (e.g., *PDE6C*, *GNAT2*, *ATF6*).^10^^–^^12^ For example, the cone mosaic has been reported as being near normal in appearance in patients with *GNAT2*-associated ACHM^11^ and nearly absent in patients with *ATF6*-associated ACHM.^12^

AOSLO has also been used to enhance our understanding of the pathophysiology of albinism. Patients with albinism have been shown to have variable foveal cone packing,^13^ with strong correlations between peak cone density, foveal outer nuclear layer (ONL) thickness, and outer segment (OS) length.^14^^,^^15^ Additionally, AOSLO has been used to demonstrate that visual acuity is not highly correlated with peak cone density, suggesting post-receptor disruptions.^16^ Although AOSLO has been used in dozens of other conditions,^17^ applications to ACHM and albinism provide some of the clearest demonstrations of the contribution of AOSLO to the elucidation of disease pathophysiology.

However, these studies only report results for subjects in whom they could obtain AOSLO images that could be quantified. The inability to quantify the cone mosaic in some AOSLO images can result from various factors that may degrade image quality. These include increased axial length and/or small pupil size (which lower the lateral resolution),^18^ media opacities (e.g., cataract),^19^ and increased eye motion.^20^^,^^21^ In ACHM and albinism, nystagmus is common, and patients also can have shorter than average axial length (Warren JZ, et al. *IOVS* 2018;59:ARVO Abstract 2156).^22^ In ACHM, previous studies have reported AOSLO data for between 33% and 83% of subjects recruited.^6^^,^^7^^,^^10^^,^^11^ Similarly, previous studies in patients with albinism have reported AOSLO data for between 31% and 83% of subjects recruited.^13^^,^^14^ Despite the challenges of acquiring, processing, and analyzing AOSLO imaging in these populations, optical coherence tomography (OCT) imaging can be acquired in nearly all patients.

Given the growing use of AOSLO in challenging populations like these, it is important to know whether conclusions being drawn are representative of the range of structural and functional manifestations of the specific disease being studied or simply a subset of subjects with characteristics that make them amenable to AOSLO imaging. To this end, we sought to test the hypothesis that visual acuity and OCT metrics of photoreceptor anatomy differ in patients with ACHM or albinism for whom AOSLO images were quantifiable compared to those for whom AOSLO images were unquantifiable.

## Methods

### Subjects

This research followed the tenets of the Declaration of Helsinki and was approved by the Institutional Review Board (IRB) at the Medical College of Wisconsin (PRO00023898). Written informed consent was obtained from all participants, and their OCT and AOSLO images were stored in an IRB-approved bank. Images and corresponding subject demographics (including genetic information) were obtained using database management software (Lattice 1.0; Translational Imaging Innovations, Inc., Hickory, NC). All subjects in the ACHM cohort had a genetically confirmed diagnosis (two pathogenic mutations in either *CNGA3* or *CNGB3*), although we did not include subjects with ACHM with outer retinal atrophy. In addition, two otherwise eligible subjects with *CNGA3*-associated ACHM were excluded due to having a controversial rhodopsin mutation (F45L).^23^ The subjects included in the albinism cohort had to have a diagnosis of confirmed albinism, likely albinism, or suspected albinism. These classifications were based on the presence of clinical features commonly associated with albinism and genetic test results. The clinical features that were assessed included nystagmus, ocular hypopigmentation (as evidenced by iris transillumination and/or macular hypopigmentation), and foveal hypoplasia. A confirmed genetic diagnosis consisted of two pathogenic mutations in a gene associated with oculocutaneous albinism (OCA; *TYR*, *OCA2*, *TYRP1*, *SLC45A2*, *SLC24A5*, or any Hermansky–Pudlack syndrome gene) or a hemizygous mutation in the ocular albinism (OA) gene *GPR143*. Subjects with confirmed albinism had at least two of the three clinical features and a confirmed genetic diagnosis. Subjects with likely albinism had all three clinical features and one pathogenic mutation in an OCA gene, whereas those with suspected albinism had all three clinical features but no pathogenic mutations in any OCA or OA gene. Subjects with any of the above albinism diagnoses but who had additional ocular pathology (e.g., macular drusen, severe myopic degeneration, color vision deficiency) were not included in this study.

For subjects with ACHM, the right eye of each subject was included in this study unless only the left eye was imaged. For subjects with albinism, if both eyes were imaged with AOSLO, the eye included in this study was the eye with subjectively better OCT image quality that also included the incipient fovea, and if only one eye was imaged by AOSLO, that eye was used. Axial length was measured using an IOL Master (Carl Zeiss Meditec, Dublin, CA). Best-corrected visual acuity (BCVA) was included for each subject, if available from the study visit or most recent referral.

### OCT Imaging

For both populations, previously acquired OCT scans—Bioptigen spectral-domain OCT (Leica Microsystems, Wetzlar, Germany) or Cirrus HD-OCT (Carl Zeiss Meditec)—were included and processed as previously reported.^6^^,^^24^ Prior to imaging, the eye of a subject was dilated using either a single drop of Cyclomydril (1% phenylephrine hydrochloride and 0.2% cyclopentolate hydrochloride) or a combination of tropicamide (1%) and phenylephrine hydrochloride (2.5%) for cycloplegia and pupillary dilation. For OCT scans acquired on the Bioptigen, horizontal or vertical line scans through the fovea (containing 80–120 frames and having nominal lengths of 5–7 mm) and volume scans having a nominal size of 3 × 3 mm or 7 × 7 mm were used for analysis.

For both populations, Bioptigen OCT scans at the fovea were processed using ImageJ (National Institutes of Health, Bethesda, MD).^25^ Line scans were registered to a reference frame using the TurboReg plugin,^26^ with the reference frame selected to include the center of the fovea. Up to 30 frames were averaged in the subjects with ACHM and up to 19 frames averaged for the subjects with albinism. When an obvious foveal depression could not be visualized in subjects with albinism, the location of the incipient fovea was based on features such as the foveal reflex (if present), extrusion of inner retinal layers, OS elongation, and/or ONL widening.^27^^–^^29^ If line scans were not of sufficient quality, could not be registered to the reference frame, or did not clearly contain the foveal center, then appropriate frame(s) from a volume scan were used for analysis. For five subjects with albinism, the volume scan was not of sufficient quality for analysis, so Cirrus OCT scans were used instead. Cirrus macular cubes (512 A-scans, 128 B-scans; nominal scan size = 6 mm × 6mm) were reviewed for image quality and location of the incipient fovea. Then, either a high-density vertical or horizontal line scan or a scan from the macular cube at the location of the incipient fovea was used for analysis.

### OCT Analysis

**Figure 1.**
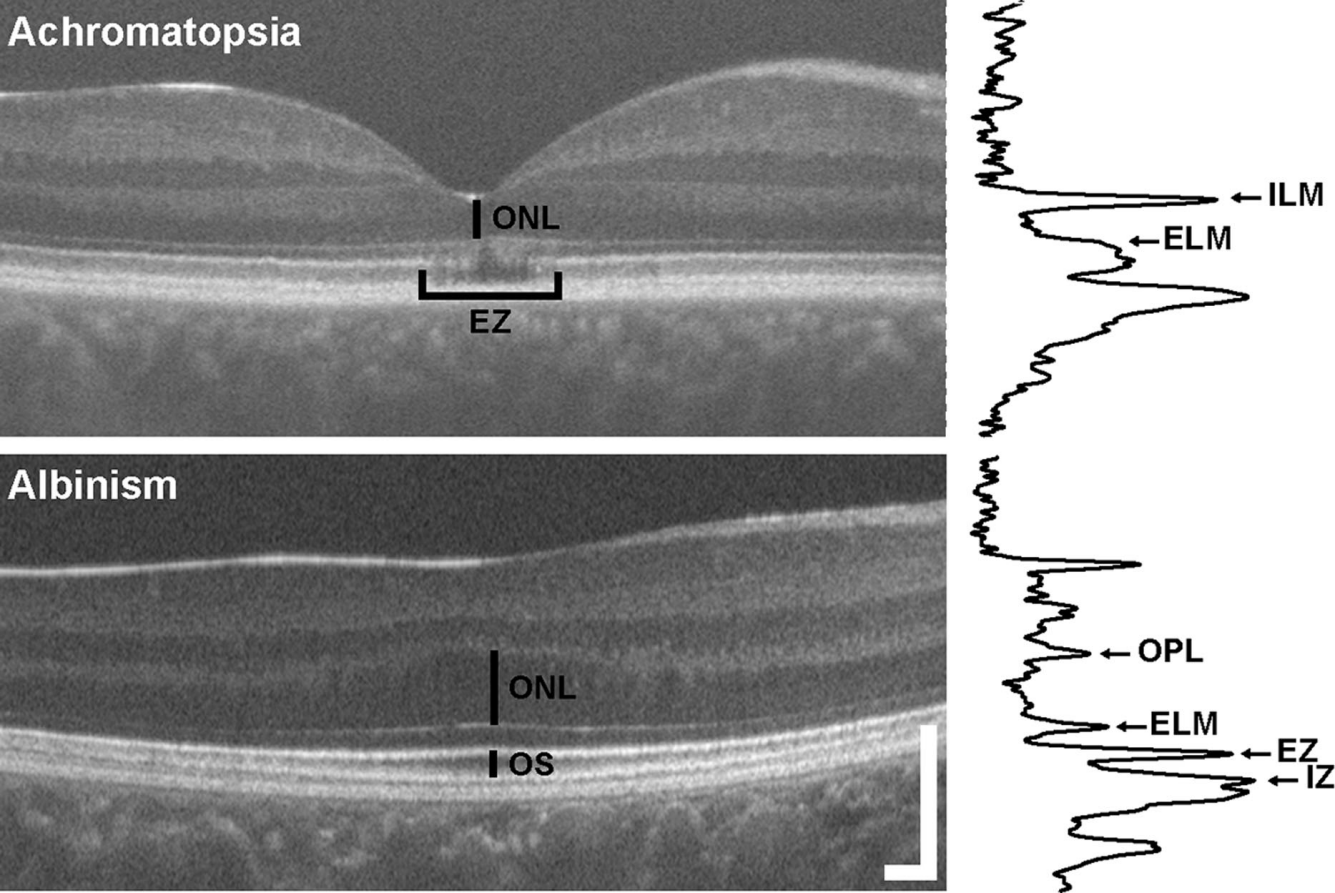
OCT-based photoreceptor metrics. For subjects with achromatopsia, foveal ONL thickness at the lowest part of the foveal pit and OCT EZ grade were assessed between the ELM and inner limiting membrane (ILM) using LRPs (example shown to right of OCT scan) in cases of complete foveal excavation as shown here. For subjects with albinism, ONL thickness was measured between the ELM and outer plexiform layer (OPL) at the location of maximum OS length. Although maximum OS length was assessed between the EZ and IZ, it is important to note for albinism that the ONL thickness and OS length measurements were not taken from the same LRP (as described in the Methods). All foveal ONL measurements included any Henle fiber layer that might have been present. *Scale bar*: 200 µm.

For subjects with ACHM, ellipsoid zone (EZ) grade was assessed by a single observer (KML) based on a previously published grading scheme,^30^ such that in grade 1 the EZ is intact and continuous; in grade 2, the EZ is disrupted; in grade 3, the EZ is absent and the external limiting membrane (ELM) is collapsed; grade 4 is a hyporeflective zone; and grade 5 is outer retinal atrophy. As those with grade 5 ACHM have, by definition, atrophied outer retinal structure at the fovea, they are not amenable to quantitative assessment and therefore could not be included in this analysis. For subjects with albinism, photoreceptor OS length was defined as the distance between the EZ and the interdigitation zone (IZ) and was measured using longitudinal reflectivity profiles (LRPs) in the linear viewing format in OCT Reflectivity Analytics (ORA).^15^ Maximum OS length was calculated by a single observer (ENW) as previously described.^15^ The location of the apparent maximum OS length was first subjectively identified by the observer (ENW), then LRPs (five pixels wide) were automatically extracted every 25 µm within 250 µm of the apparent maximum, generating a total of 21 LRPs for each OCT image. The peaks corresponding to the EZ and IZ in each LRP were labeled by the user and exported from ORA. Using MATLAB (MathWorks, Natick, MA), a single-term Gaussian function was fit to the peak-to-peak distances between the EZ and IZ as a function of retinal location. The maximum of the fitted function was used to calculate both the maximum OS length and the location of the incipient fovea in each scan (Fig. 1).

For both populations, the foveal ONL thickness included both the true ONL plus Henle fiber layer and was measured by a single observer (KML) using LRPs in the ORA software.^15^^,^^24^ For subjects with ACHM, foveal ONL thickness was measured at the lowest part of the foveal pit; for subjects with albinism, foveal ONL thickness was measured at the location of maximum OS length (as a marker of the incipient fovea, due to variable foveal pit morphology in the albinism population) (Fig. 1). The boundaries of the ONL were selected from the peaks of the LRP corresponding to the inner limiting membrane and ELM in cases of complete foveal excavation and from the posterior boundary of the outer plexiform layer to the peak of the ELM in cases of foveal hypoplasia.

For subjects with ACHM, foveal OCT scans were reviewed by a single observer (KML) to determine the presence of foveal hypoplasia (defined as persistence of at least one inner retinal layer through the fovea). For subjects with albinism, foveal hypoplasia grade according to the Leicester system^29^ was subjectively assessed independently by two observers (ENW and NW) to describe the presence or absence of a foveal pit, extrusion of inner retinal layers, ONL widening, and OS lengthening. For scans where the two observers disagreed, they reviewed and discussed the foveal features of those scans to arrive at a consensus grade, which was used as the final foveal hypoplasia grade.

### AOSLO Imaging and Analysis

Subjects included in this study had AOSLO imaging previously attempted on at least one eye. If image quality was sufficient, AOSLO videos were processed into single TIF images and montaged, as previously reported.^6^^–^^9^^,^^13^^,^^16^ An experienced observer assessed non-waveguiding remnant cone structure in split-detector AOSLO images and montages, if available, for subjects with ACHM (KML) and waveguiding cones in confocal AOSLO images for subjects with albinism (ENW). Quantifiable AOSLO images contained the peak cone density at the fovea that could be assessed, and unquantifiable AOSLO images were images where cones at the fovea could not be resolved. As the reliance on the quantification of foveal cones restricts the use of AOSLO data, we did a secondary analysis where subject classification included the parafoveal mosaic. Usable AOSLO images contained the location of the peak cone density that could be inferred from a montage based on features such as the rod-free zone for ACHM and cone packing for albinism, and the resolution of images in the parafoveal mosaic was of sufficient quality to assess cones quantitatively. Unusable AOSLO images were images where parafoveal cones could not be resolved. Any AOSLO dataset was classified as unquantifiable or unusable if the images could not be processed or montaged because of poor image quality, often limited by motion artifacts caused by nystagmus or poor optics of the eye. Subjects were grouped by AOSLO status for comparison of BCVA and OCT metrics, as described above.

### Statistical Analyses

Statistical analyses were done using Prism 9 (GraphPad Software, La Jolla, CA). The Shapiro–Wilk normality test was used to determine the use of parametric or nonparametric tests when appropriate. As our hypothesis testing relates to the fovea, reported *P* values are adjusted within each condition using the Holm method to account for the family-wise error rate when performing multiple statistical tests.^31^ Descriptive group comparison of age was not adjusted.

## Results

### Subject Demographics

We included a total of 84 subjects with ACHM (41 female, 43 male) and 82 subjects with albinism (41 female, 41 male) in this study. The albinism cohort included 52 subjects with confirmed albinism (63.4%), 24 subjects with likely albinism (29.3%), and six subjects with suspected albinism (7.3%). The mean ± SD age was 23.7 ± 13.5 years (range, 7–64 years) for subjects with ACHM and 20.0 ± 13.1 years (range, 5–69 years) for subjects with albinism. The mean ± SD axial length was 23.97 ± 1.96 mm for subjects with ACHM and 22.97 ± 1.90 mm for subjects with albinism, compared to 24.08 ± 1.05 mm for previously reported controls.^32^ Seventy-six of the subjects with ACHM have been previously reported elsewhere (Supplementary Table S1),^4^^,^^6^^–^^9^^,^^12^^,^^24^^,^^33^^–^^37^ and 56 of the subjects with albinism have been previously reported elsewhere (Supplementary Table S2).^13^^–^^16^^,^^32^ Genetic information for the 34 subjects who have not been included in previous studies is provided in Supplementary Table S3.

### AOSLO Success Rates

**Figure 2.**
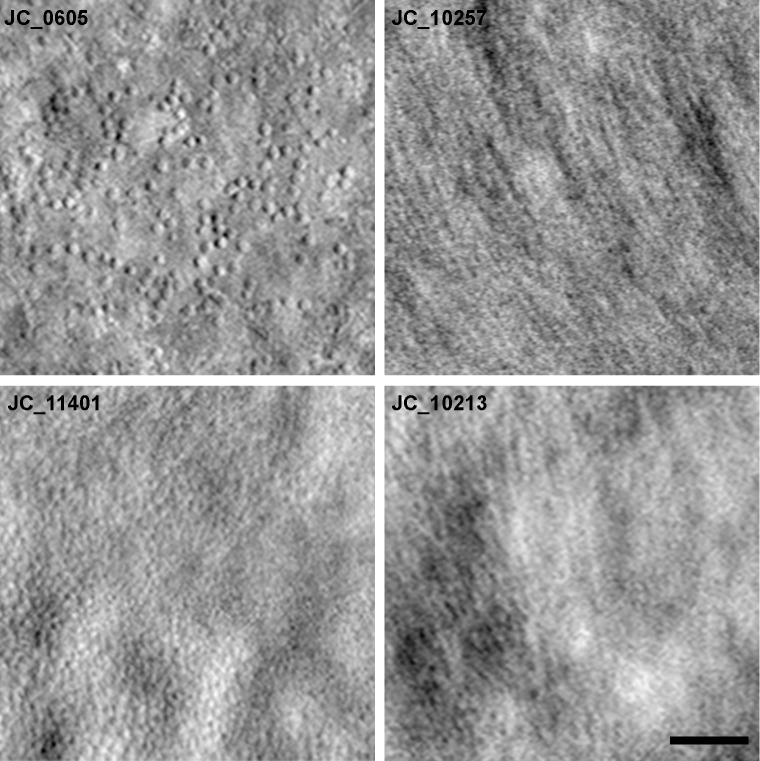
Examples of variable AOSLO image quality in subjects with achromatopsia. Shown are split-detector images demonstrating variability in images that are quantifiable or usable (JC_0605 and JC_11401) versus images that are unquantifiable (JC_10257 and JC_10213). Although the peak cone density in JC_11401 cannot be accurately quantified due to packing of cones and resolution of split-detector imaging, parafoveal cones are easily identifiable and allow estimation of the location of peak cone density. *Scale bar*: 50 µm.

**Figure 3.**
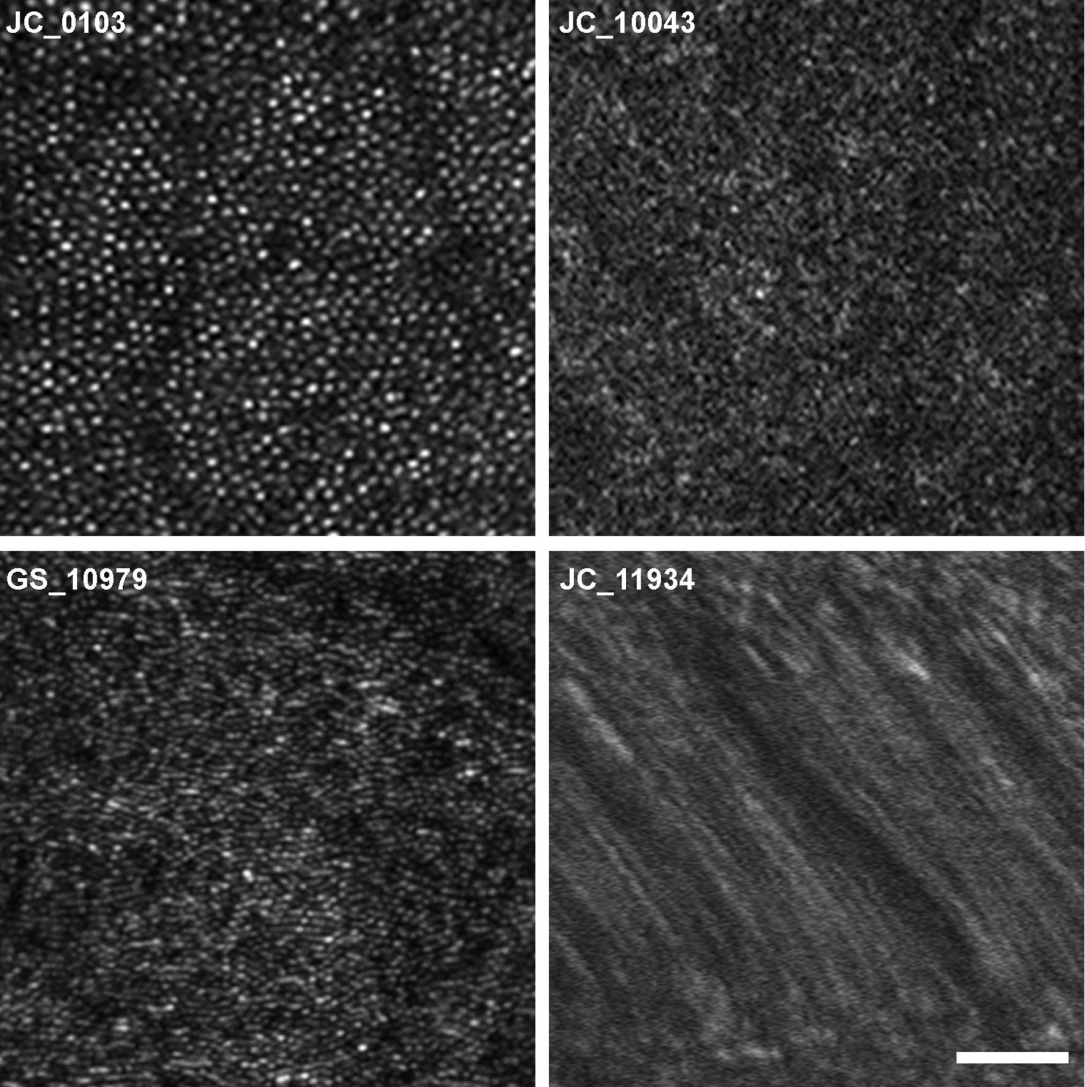
Examples of variable AOSLO image quality in subjects with albinism. Shown are confocal images demonstrating variability in images that are quantifiable (JC_0103 and GS_10979) versus images that are unquantifiable (JC_10043 and JC_11934). *Scale bar*: 50 µm.

In our ACHM cohort, 49% (41/84) of subjects had AOSLO images where the peak foveal cone density could be quantified. Examples of quantifiable and unquantifiable split-detector AOSLO images are shown in Figure 2. In our albinism cohort, 57% (47/82) of subjects had quantifiable AOSLO images where the peak foveal cone density could be quantified. Examples of quantifiable and unquantifiable confocal AOSLO images are shown in Figure 3. As the location of the peak foveal cone density can be used to guide parafoveal analyses even if the precise foveal cone density value cannot be determined, we also used an additional definition of success. Here, an AOSLO montage was considered usable if the parafoveal cones were clearly resolved and permitted estimation of the location of peak cone density (e.g., JC_11401 in Fig. 2). By these criteria, 60% (50/84) of subjects with ACHM were classified as having usable AOSLO images, and 62% (51/82) of subjects with albinism were classified as having usable AOSLO images.

In the ACHM cohort, subjects with quantifiable AOSLO images were older (mean ± SD = 26.4 ± 12.9 years) than those with unquantifiable AOSLO images (mean ± SD = 21.2 ± 13.8 years; *P* = 0.0214, Mann–Whitney test). Subjects with ACHM and quantifiable AOSLO images were between 9 and 64 years of age (including 12 children), and subjects with unquantifiable AOSLO images were between 7 and 57 years of age (including 23 children). Using the AOSLO success criteria that included the parafoveal mosaic, subjects with ACHM and usable AOSLO images were older (mean ± SD = 25.5 ± 12.9 years) than those with unusable AOSLO images (mean ± SD = 21.1 ± 14.2 years), although this trend was not significant (*P* = 0.0503, Mann–Whitney test).

In the albinism cohort, subjects with quantifiable AOSLO images were significantly older (mean ± SD = 23.0 ± 13.9 years) than those with unquantifiable AOSLO images (mean ± SD = 16.1 ± 11.1 years; *P* = 0.0073, Mann–Whitney test). Subjects with albinism and quantifiable AOSLO images were between 6 and 69 years of age (including 22 children), and subjects with unquantifiable AOSLO images were between 5 and 54 years of age (including 24 children). Using the AOSLO success criteria that included the parafoveal mosaic, subjects with albinism and usable AOSLO images were significantly older (mean ± SD = 22.7 ± 13.3 years) than those with unusable AOSLO images (mean ± SD = 15.6 ± 11.7 years; *P* = 0.0014, Mann–Whitney test).

### Best-Corrected Visual Acuity

**Figure 4.**
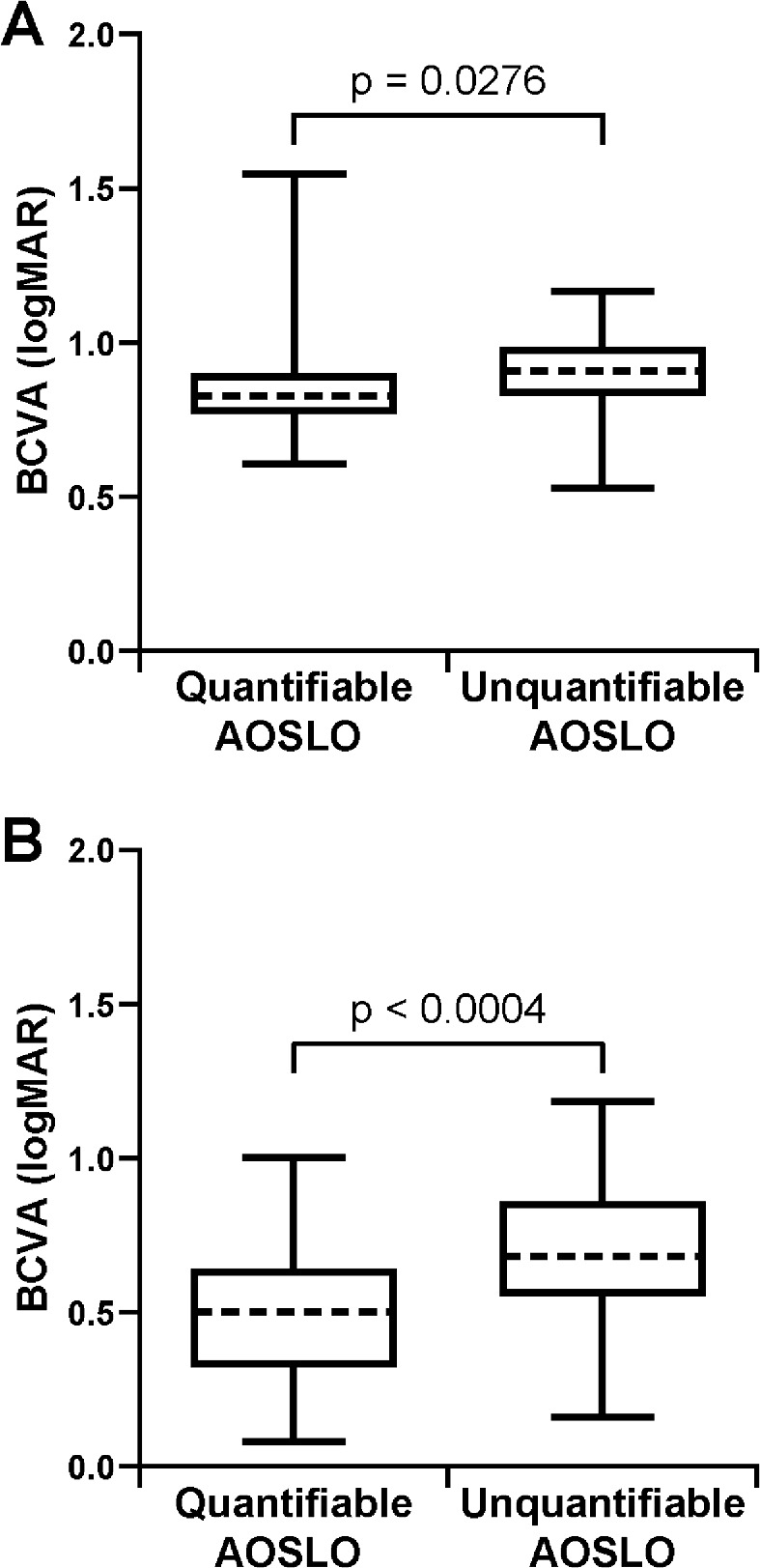
Subjects with quantifiable AOSLO images have significantly better BCVA than subjects with unquantifiable AOSLO images. This trend was present in both (**A**) subjects with achromatopsia (*P* = 0.0276, Mann–Whitney test) and (**B**) subjects with albinism (*P* < 0.0004, unpaired *t*-test). The *ends of the boxes* are the 25th and 75th percentiles, the *dashed line* is the median, and the *whiskers* span the range of data.

BCVA was available for 83 of 84 subjects with ACHM. As shown in Figure 4A, subjects with ACHM and quantifiable AOSLO images had significantly better BCVA (mean ± SD = 0.84 ± 0.15 logMAR; Snellen equivalent range, 20/80 to 20/800) than those with unquantifiable AOSLO images (mean ± SD = 0.89 ± 0.13 logMAR; Snellen equivalent range, 20/63 to 20/320), although there was overlap between the two groups (*P* = 0.0276, Mann–Whitney test). Using the AOSLO success criteria that included the parafoveal mosaic, subjects with ACHM and usable AOSLO images again had significantly better BCVA than those with unusable AOSLO images (mean ± SD = 0.84 ± 0.15 vs. 0.91 ± 0.12 logMAR, respectively; *P* = 0.0072, Mann–Whitney test) (Supplementary Fig. S1A).

BCVA was available for 80 of 82 subjects with albinism. As shown in Figure 4B, subjects with albinism and quantifiable AOSLO images had significantly better BCVA (mean ± SD = 0.48 ± 0.21 logMAR; Snellen equivalent range, 20/25+1 to 20/200) than those with unquantifiable AOSLO images (mean ± SD = 0.70 ± 0.25 logMAR; Snellen equivalent range, 20/32+2 to 20/320+1), although there was again overlap between the two groups (*P* < 0.0004, unpaired *t*-test). Using the AOSLO success criteria that included the parafoveal mosaic, subjects with albinism and usable AOSLO images again had significantly better BCVA than those with unusable AOSLO images (mean ± SD = 0.50 ± 0.21 vs. 0.69 ± 0.26 logMAR, respectively; *P* = 0.0040, unpaired *t*-test) (Supplementary Fig. S1B).

### Foveal Outer Retinal Structure

**Figure 5.**
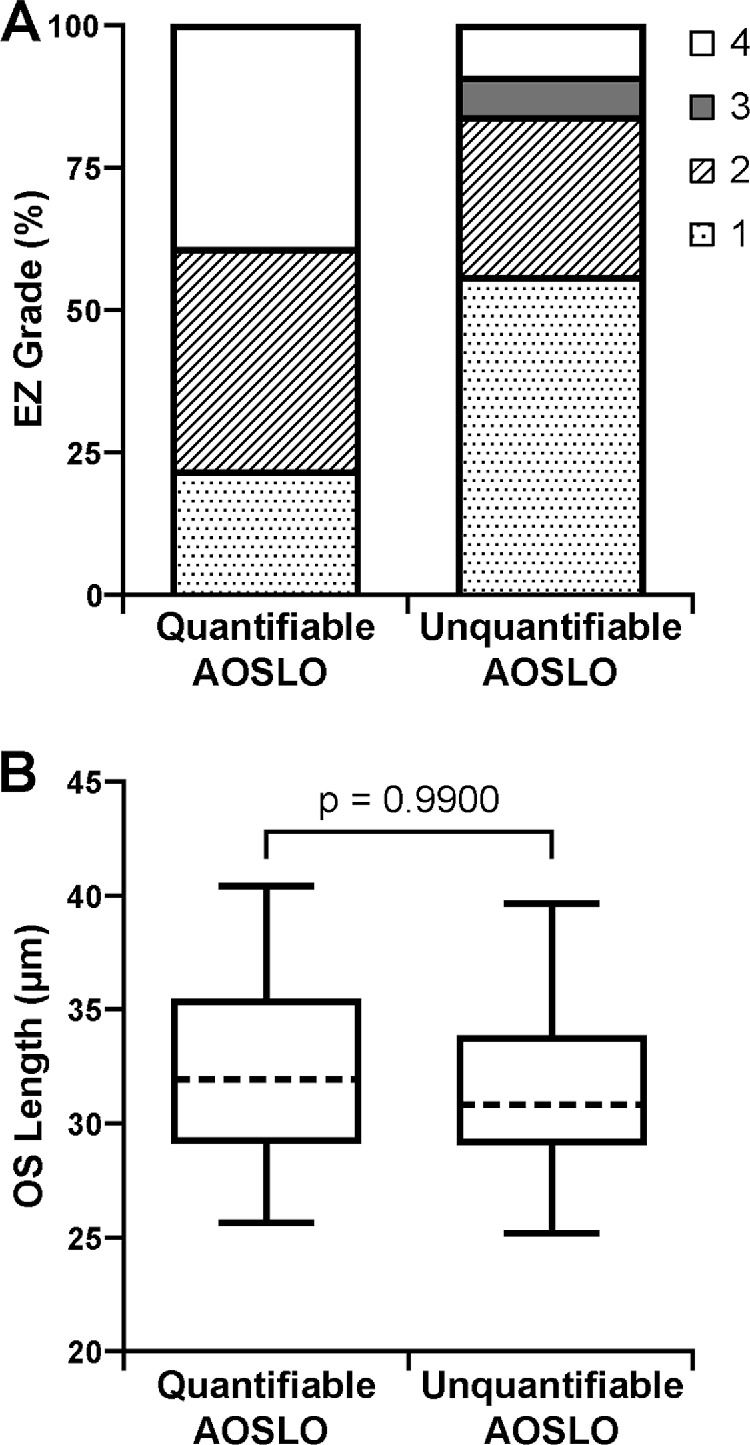
Representation of foveal outer retinal structure in subjects with quantifiable AOSLO images versus subjects with unquantifiable AOSLO images. (**A**) For achromatopsia, the EZ was graded as grade 1 if the EZ was intact and continuous, grade 2 if the EZ was disrupted, grade 3 if the EZ was absent and the external limiting membrane was collapsed, and grade 4 if a hyporeflective zone was present.^30^ There was a significant trend between the appearance of the EZ and the ability to quantify AOSLO images (*P* = 0.0028, χ^2^ test for trend). (**B**) For albinism, there was no significant difference in OS length between those subjects with albinism for whom AOSLO images were quantifiable (mean ± SD = 32.32 ± 3.82 µm) and those for whom AOSLO images were unquantifiable (mean ± SD = 31.41 ± 3.71 µm; *P* = 0.9900, Mann–Whitney test). The *ends of the boxes* are the 25th and 75th percentiles, the *dashed line* is the median, and the *whiskers* span the range of data.

As described in the methods, the integrity of foveal cones in the outer retina was evaluated in subjects with ACHM using a previously described EZ grading system.^30^ Across the 84 subjects where the EZ could be graded, 33 subjects (39%) had a grade 1 EZ, 28 subjects (33%) had a grade 2 EZ, three subjects (4%) had a grade 3 EZ, and 20 subjects (24%) had a grade 4 EZ; this distribution was generally consistent with observations in other ACHM cohorts.^30^^,^^38^ For EZ grade, there was a significant trend between the appearance of the EZ and the ability to quantify AOSLO images (*P* = 0.0028, χ^2^ test for trend) (Fig. 5A). Interestingly, in the ACHM cohort, 56% of subjects with unquantifiable AOSLO images had a grade 1 EZ, whereas only 22% of subjects with quantifiable AOSLO images had a grade 1 EZ. In addition, 9% of subjects with unquantifiable AOSLO images had a grade 4 EZ, whereas 39% of subjects with quantifiable AOSLO images had a grade 4 EZ. When using the AOSLO success criteria that included the parafoveal mosaic, there was again a trend between the EZ grade and the usability of the AOSLO images (*P* = 0.0126, χ^2^ test for trend) (Supplementary Fig. S2A).

Because the EZ is intact in all subjects with albinism and there is an established association between OS length and cone density in albinism,^15^ we examined the foveal OS length in the 81 subjects with analyzable OCT scans. As shown in Figure 5B, there was no significant difference in OS length between subjects with albinism having quantifiable AOSLO images (mean ± SD = 32.32 ± 3.82 µm) and those with unquantifiable AOSLO images (mean ± SD = 31.41 ± 3.71 µm; *P* = 0.9900, Mann–Whitney test). This did not change when using the AOSLO success criteria that included the parafoveal mosaic, with the subjects with albinism and usable AOSLO images having a mean ± SD OS length of 32.03 ± 3.82 µm and those with unusable AOSLO images having a mean ± SD OS length of 31.77 ± 3.72 µm (*P* = 1, Mann–Whitney test) (Supplementary Fig. S2B).

### Foveal Outer Nuclear Layer Thickness

**Figure 6.**
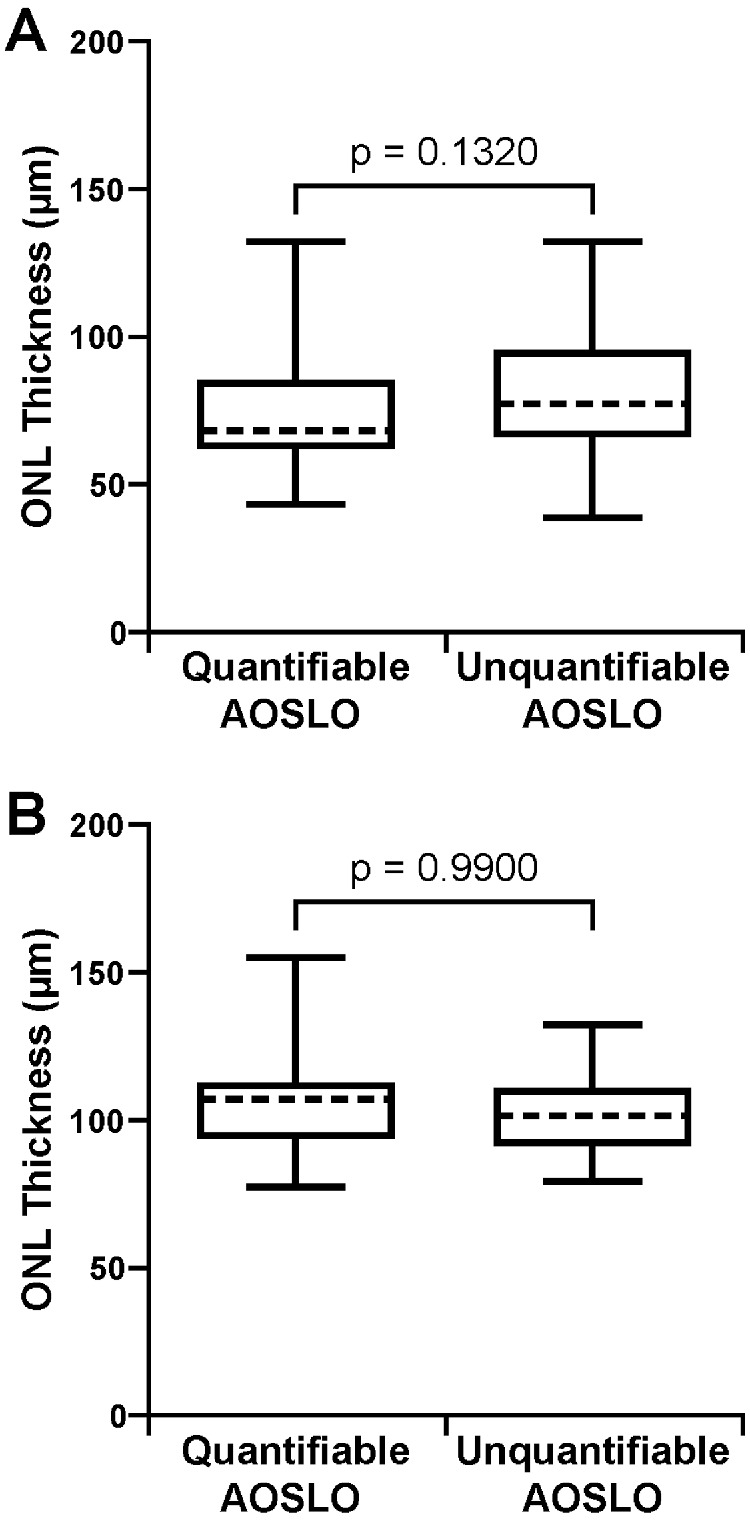
Foveal ONL thickness was not significantly different in subjects with quantifiable AOSLO images compared to subjects with unquantifiable AOSLO images. This was the case in both (**A**) subjects with achromatopsia (*P* = 0.1320, Mann–Whitney test) and (**B**) subjects with albinism (*P* = 0.9900, unpaired *t*-test). The *ends of the boxes* are the 25th and 75th percentiles, the *dashed line* is the median, and the *whiskers* span the range of data.

In the ACHM cohort, subjects with quantifiable AOSLO images had a thinner ONL (mean ± SD = 72.62 ± 18.19 µm) compared to those with unquantifiable AOSLO images (mean ± SD = 79.50 ± 20.28 µm), although this difference was not significant (*P* = 0.1320, Mann–Whitney test) (Fig. 6A). When using the AOSLO success criteria that included the parafoveal mosaic, subjects with ACHM and usable AOSLO images again had a thinner ONL (mean ± SD = 72.68 ± 18.78 µm) compared with subjects with unusable AOSLO images (mean ± SD = 81.23 ± 19.65 µm), and this difference was not significant (*P* = 0.0666, Mann–Whitney test) (Supplementary Fig. S3A).

ONL thickness values were available for 81 of the 82 subjects with albinism. As shown in Figure 6B, there was no significant difference in ONL thickness between subjects with quantifiable AOSLO (mean ± SD = 104.78 ± 16.23 µm) and those with unquantifiable AOSLO in the albinism cohort (mean ± SD = 101.42 ± 13.70 µm; *P* = 0.9900, unpaired *t*-test). This did not change when using the AOSLO success criteria that included the parafoveal mosaic, with the subjects with albinism and usable AOSLO images having a mean ± SD ONL thickness of 103.89 ± 16.06 µm and subjects with unusable AOSLO images having a mean ± SD ONL thickness of 102.50 ± 13.91 µm (*P* = 1, Mann–Whitney test) (Supplementary Fig. S3B).

### Foveal Hypoplasia

**Figure 7.**
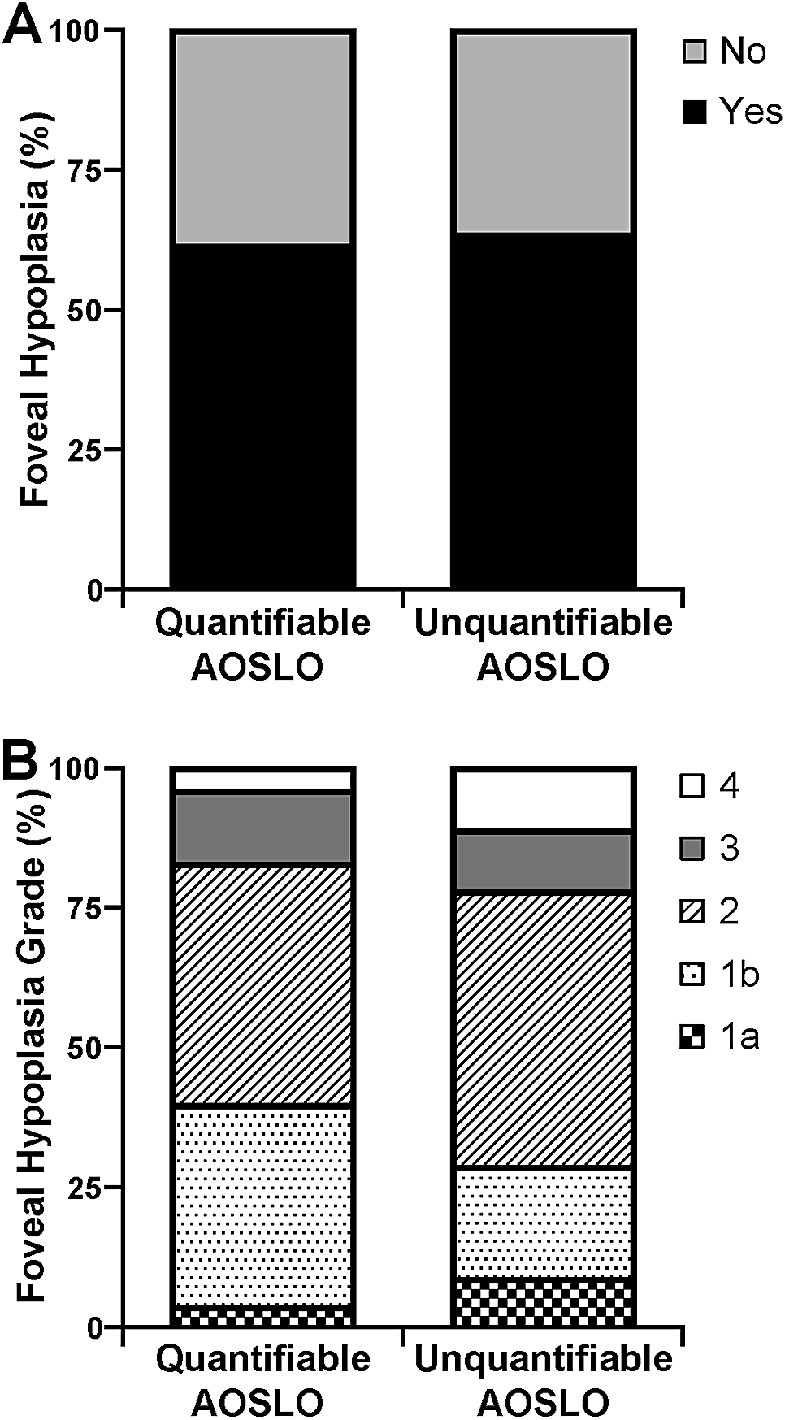
Foveal hypoplasia assessment was not significantly different in subjects with quantifiable AOSLO images compared with subjects with unquantifiable AOSLO images. This trend was present in both (**A**) subjects with achromatopsia for the presence or absence of foveal hypoplasia (*P* > 0.9999, Fisher's exact test) and (**B**) subjects with albinism for foveal hypoplasia grade according to the Leicester system^29^ (*P* = 1, χ^2^ test for trend). For albinism, foveal hypoplasia grade 1a is marked by the absence of the extrusion of plexiform layers and presence of a nearly normal foveal pit, OS lengthening, and ONL widening; grade 1b is absence of the extrusion of plexiform layers and presence of a shallow foveal pit, OS lengthening, and ONL widening; grade 2 is absence of the extrusion of plexiform layers and foveal pit and presence of OS lengthening and ONL widening; grade 3 is absence of the extrusion of plexiform layers, foveal pit, and OS lengthening and presence of ONL widening; and grade 4 is absence of the extrusion of plexiform layers, foveal pit, OS lengthening, and ONL widening.

Foveal hypoplasia is a common feature seen in patients with ACHM,^30^ and its assessment in ACHM as the presence or absence of at least one inner retinal layer through the fovea does not take into account features of the outer retina like foveal hypoplasia grading in albinism. As shown in Figure 7A, the presence of foveal hypoplasia in ACHM was not significantly different in subjects with quantifiable AOSLO images compared with subjects with unquantifiable AOSLO images (*P* > 0.9999, Fisher's exact test). This trend did not change when using the AOSLO success criteria that included the parafoveal mosaic (*P* > 0.9999, Fisher's exact test) (Supplementary Fig. S4A).

As described in Methods, foveal hypoplasia grade according to the Leicester system^29^ was assessed in subjects with albinism as it includes features of the outer retina (i.e., ONL widening and OS elongation). As shown in Figure 7B, there was no significant trend between the presence or absence of a foveal pit, extrusion of inner retinal layers, ONL widening, and OS lengthening and the ability to quantify AOSLO images in the albinism cohort (*P* = 0.9900, χ^2^ test for trend). This trend did not change when using the AOSLO success criteria that included the parafoveal mosaic (*P* = 1, χ^2^ test for trend) (Supplementary Fig. S4B).

## Discussion

In this study, we compared metrics of foveal structure and function between subjects for whom AOSLO images could be quantified or used and for whom AOSLO images could not be quantified or used in both ACHM and albinism. For albinism, there were no differences in any structural metrics in our population likely to contain good imaging candidates, suggesting that cone density data reported in albinism are likely an accurate representation of cone topography in the condition despite about 40% of subjects with albinism being classified as having unquantifiable or unusable AOSLO images. In contrast, we observed differences in EZ grade in the ACHM cohort. This was unexpected, as a previous study showed age and BCVA not to be different across the EZ grades, including EZ grade 5.^30^ Paradoxically, we found that in the ACHM cohort, subjects with quantifiable AOSLO images had a worse EZ grade than those with unquantifiable AOSLO images. It is important to note that there is conflicting evidence about the relationship between EZ grade and foveal cone density in ACHM.^6^^,^^7^ One study with subjects with *CNGA3*-associated ACHM showed no difference in peak cone density between subjects with EZ grades 2 and 4 but had too few subjects with EZ grades 1 and 3 to include for the statistical analysis. Conversely, another study with subjects with *CNGB3*-associated ACHM showed a significant association between EZ grades, including subjects with EZ grade 1, and peak cone density, despite also having too few subjects with EZ grade 3 to include for the statistical analysis. Therefore, it is not clear whether EZ differences in our study are suggestive of better or worse remnant cone structure in the subjects with unquantifiable AOSLO images. In addition, the 10% difference in ONL thickness observed when using the AOSLO success criteria that included the parafoveal mosaic, although not significant (*P* = 0.0666), was in the direction of subjects with usable AOSLO images having a thinner ONL. The measured ONL thickness can be affected by EZ disruptions, which can cause an elevation of the ELM and thus an apparent thinning of the ONL. As well, the ONL may contain variable amounts of the Henle fiber layer; thus, it is difficult to rely on ONL thickness as an accurate surrogate of remnant cone structure in ACHM. Indeed, previous studies have reported no correlation between foveal ONL thickness and peak foveal cone density in patients with *CNGA3*-associated ACHM.^7^ Nevertheless, our observed differences in OCT structure between subjects with ACHM for whom AOSLO images were quantifiable/usable and those for whom images were unquantifiable/unusable suggest that caution should be taken in generalizing prior AOSLO-based reports of remnant cone structure in ACHM to the disease as a whole.

Separate from metrics of photoreceptor structure, we observed interesting results for BCVA. For both populations, subjects with quantifiable or usable AOSLO images had better BCVA than subjects with unquantifiable or unusable AOSLO images (there was significant overlap in BCVA between the groups within both the ACHM and albinism cohorts). The direction and magnitude of the difference are consistent with those reported previously for 66 subjects with *CNGA3*- or *CNGB3*-associated ACHM (compared to 84 subjects here), although that difference was not statistically significant (*P* = 0.07).^37^ Although these differences are important to be aware of, they likely do not portend underlying differences in cone structure. Visual acuity has been shown to be an insensitive measure of cone structure, as cone density must decrease about 40% before a clinically measurable change can be detected in visual acuity.^39^ In addition, in subjects with albinism, it has been shown that BCVA does not correlate with peak cone density.^13^ There are likely other optical and neural factors contributing to the reduced visual acuity in these populations. It is also important to note that some subjects with nystagmus often adopt an anomalous head posture,^40^ which they may use when assessing BCVA. These anomalous head postures are not accommodated by the patient interface (dental impression) of the AOSLO device used here, so this may confound some of the comparisons between BCVA and AOSLO imaging success. Similarly, it is important to note that, although the visual acuity included for analysis is best corrected, the retinal imaging is often performed using focus settings that optimize the retinal image quality and thus could result in suboptimal fixation during imaging. That said, our data appear consistent with the idea that subjects with ACHM or albinism who have better visual acuity could be better candidates for obtaining usable AOSLO images.

In both the ACHM and albinism cohorts, subjects with unquantifiable AOSLO images were younger than those with quantifiable AOSLO images. This is not surprising because nystagmus amplitude tends to diminish with age in both ACHM^9^^,^^41^ and albinism.^42^ Because of this, it is likely that an older population of subjects might yield a higher AOSLO success rate compared to a younger population of subjects. However, the age differences reported here likely do not predict differences in cone structure between the two AOSLO groups in either the ACHM or albinism cohorts. First, we are not aware of any reports of disease progression in albinism (though these patients would presumably be subject to age-related vision problems that occur in the population at large, such as cataracts and age-related macular degeneration). Second, ACHM is generally thought to be a stable condition,^6^^,^^9^^,^^30^^,^^43^^–^^45^ although there are some reports of progression in some patients.^7^^,^^46^^,^^47^ As such, like the BCVA findings, we think the age differences are perhaps misleading with regards to our central question of differences in foveal structure. Here, older subjects are likely just better candidates for AOSLO imaging—not because of any underlying differences in cone structure but because of other unrelated factors (e.g., reduced nystagmus with increasing age, improved attention span).

Our study has some important limitations. First, many of the subjects included in this study were referred and likely to be cooperative and good imaging candidates. This may be an additional bias to consider because patients with severe nystagmus or media opacities would suggest to the investigator that they would not be good imaging candidates. As these patients would not be included in this study, our reported percentage of subjects for whom AOSLO images are unusable may be an underestimate of that for the albinism and ACHM populations as a whole. This is important to consider when designing clinical trials involving AOSLO in these populations. Next, we used different observers for many of the structural measurements. These observers were experts in the population and imaging modality they assessed, and we believe this benefits the data compared with having one observer assess all measurements across the different conditions. In addition, these findings may only apply to the Medical College of Wisconsin AOSLO system without eye tracking. AOSLO systems with eye tracking^48^ or increased image acquisition speed^49^ may increase the AOSLO success rate in these patient populations, making it possible to capture a larger range of differences in cone structure within a condition. Moreover, the ability to quantify the photoreceptor mosaic or use a montage may be limited by the AOSLO modality. Confocal images are suitable for use in albinism, where the waveguide property of the cones remains intact,^13^ but split-detector images are required in ACHM to reveal non-waveguiding cones.^4^ Compared to split-detector images, confocal images have higher lateral resolution, allowing higher-density foveal cone mosaics to be quantified. Consistent with this, there were nine subjects with ACHM but only four subjects with albinism who had unquantifiable AOSLO images due to having unresolvable cones at the foveal center (but who all had an otherwise intact AOSLO montage and good image quality in the parafovea, deemed usable with the more relaxed grading criteria). It is thus possible that reported foveal cone density values may, on average, slightly underestimate that for the ACHM population as a whole. It is important for studies to define their success criteria for imaging, as well as the reasons for failure (image quality or a foveal cone mosaic below the resolution limit of the AOSLO system).

There are other uses for an AOSLO montage than only reporting photoreceptor metrics. Our first analysis relied on quantifying peak cone density, as this has been used in previous studies of ACHM and albinism.^6^^–^^9^^,^^13^^,^^15^^,^^16^^,^^35^ Although we focused on photoreceptor structure in ACHM and albinism, comparing subjects in other populations for whom AOSLO images are not always quantifiable or usable will be important, especially in future AOSLO studies examining other retinal structures (e.g., vessels, blood cells, retinal pigment epithelium). As AOSLO becomes more accessible for both research and clinical applications it is imperative that we understand its practical limitations. Because AOSLO is not successful for every patient, we show that the data may not represent the full spectrum of retinal structure for all diseases. This directly influences data interpretation for past studies and may also inform improvement in the technical and methodological design of future studies aimed at understanding disease pathophysiology.

## Supplementary Material

Supplement 1

Supplement 2

Supplement 3

Supplement 4

Supplement 5

Supplement 6

Supplement 7
