# Supplementary material for: Examining Whether AOSLO-Based Foveal Cone Metrics in Achromatopsia and Albinism Are Representative of Foveal Cone Structure
Source: Transl Vis Sci Technol. 2021 May 17;10(6):22. doi: 10.1167/tvst.10.6.22 (PMC8132001; doi:10.1167/tvst.10.6.22)
Supplement: Supplement 1 [file tvst-10-6-22_s001.pdf]

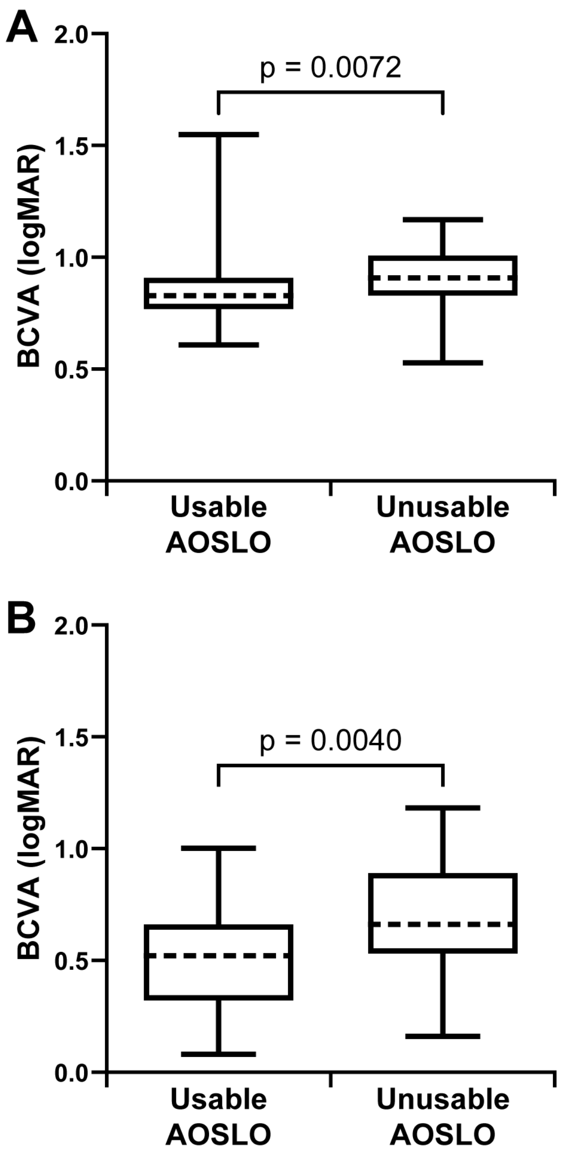

Supplementary Figure S1. Subjects with usable AOSLO images have significantly better BCVA than subjects with unusable AOSLO images. This trend was present in both (A) subjects with achromatopsia ($p = 0.0072$, Mann-Whitney test) and (B) subjects with albinism ($p = 0.0040$, unpaired t-test). The ends of the boxes are the 25th and 75th percentiles, the dashed line is the median, and the whiskers span the range of data.
