# Supplementary material for: Examining Whether AOSLO-Based Foveal Cone Metrics in Achromatopsia and Albinism Are Representative of Foveal Cone Structure
Source: Transl Vis Sci Technol. 2021 May 17;10(6):22. doi: 10.1167/tvst.10.6.22 (PMC8132001; doi:10.1167/tvst.10.6.22)
Supplement: Supplement 2 [file tvst-10-6-22_s002.pdf]

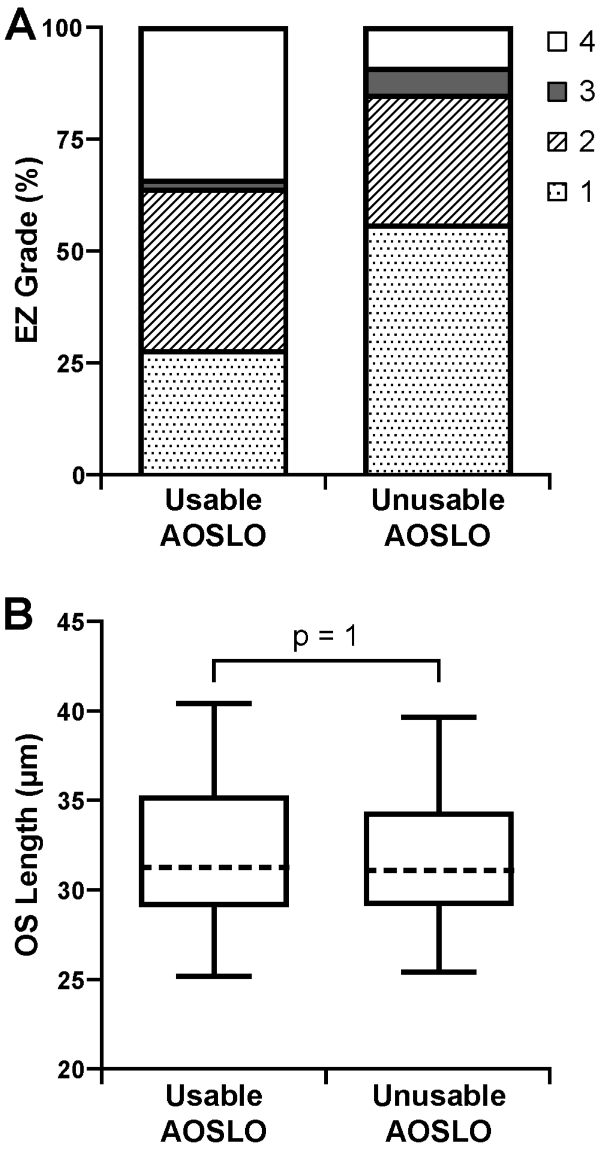

Supplementary Figure S2. Representation of foveal outer retinal structure in subjects with usable AOSLO images versus subjects with unusable AOSLO images. (A) For achromatopsia, the ellipsoid zone (EZ) was graded as grade 1 if the EZ was intact and continuous, grade 2 if the EZ was disrupted, grade 3 if the EZ was absent and the external limiting membrane was collapsed, and grade 4 if a hyporeflective zone was present. There was a significant trend between the appearance of the EZ and the ability to quantify AOSLO images (p = 0.0126, Chi-square test for trend). (B) For albinism, there was no significant difference in OS length between those subjects with albinism for whom AOSLO images were usable (mean ± SD = 32.03 ± 3.82 µm) and those for whom AOSLO images were unusable (mean ± SD = 31.77 ± 3.72 µm; p = 1, Mann-Whitney test). The ends of the boxes are the 25th and 75th percentiles, the dashed line is the median, and the whiskers span the range of data.
