# Supplementary material for: Examining Whether AOSLO-Based Foveal Cone Metrics in Achromatopsia and Albinism Are Representative of Foveal Cone Structure
Source: Transl Vis Sci Technol. 2021 May 17;10(6):22. doi: 10.1167/tvst.10.6.22 (PMC8132001; doi:10.1167/tvst.10.6.22)
Supplement: Supplement 3 [file tvst-10-6-22_s003.pdf]

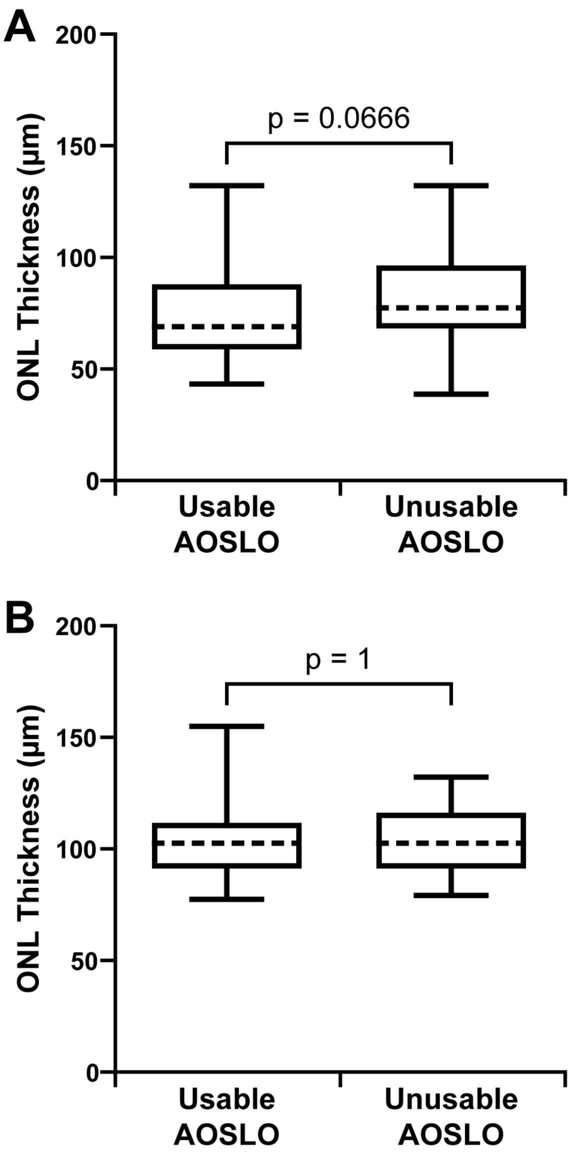


**Supplementary Figure S3.** Foveal outer nuclear layer (ONL) thickness was not significantly different in subjects with usable AOSLO images compared to subjects with unusable AOSLO images. This trend was present in both (A) subjects with achromatopsia ($p = 0.0666$, Mann-Whitney test) and (B) subjects with albinism ($p = 1$, unpaired t-test). The ends of the boxes are the 25th and 75th percentiles, the dashed line is the median, and the whiskers span the range of data.
