# Supplementary material for: Examining Whether AOSLO-Based Foveal Cone Metrics in Achromatopsia and Albinism Are Representative of Foveal Cone Structure
Source: Transl Vis Sci Technol. 2021 May 17;10(6):22. doi: 10.1167/tvst.10.6.22 (PMC8132001; doi:10.1167/tvst.10.6.22)
Supplement: Supplement 4 [file tvst-10-6-22_s004.pdf]

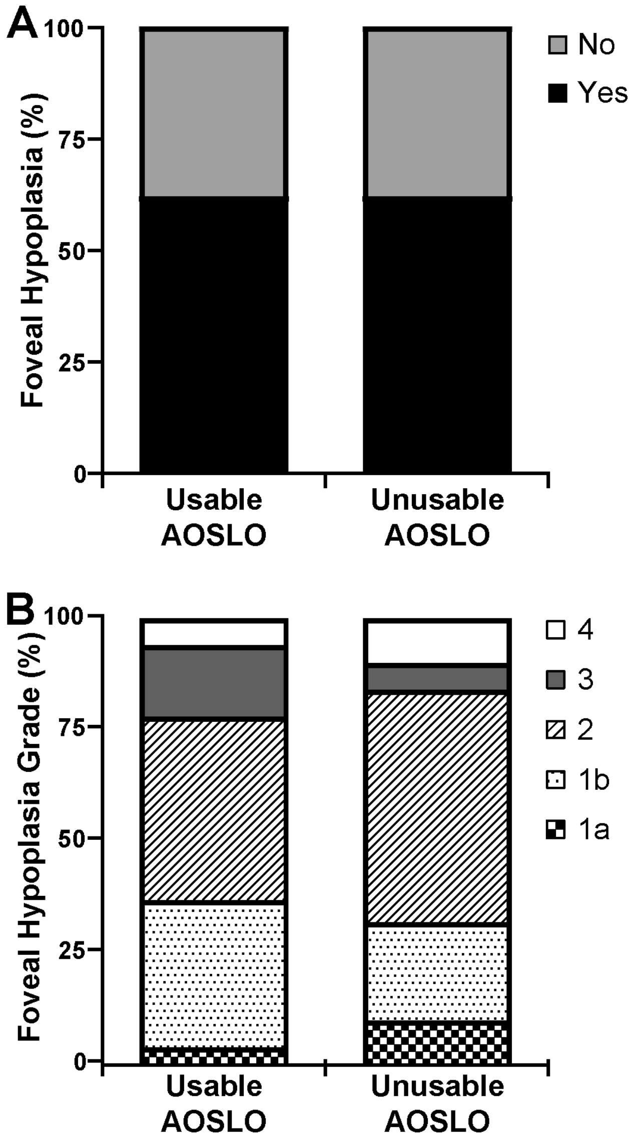

Supplementary Figure S4. Foveal hypoplasia assessment was not significantly different in subjects with usable AOSLO images compared to subjects with unusable AOSLO images. This trend was present in both (A) subjects with achromatopsia for the presence or absence of foveal hypoplasia ($p > 0.9999$, Fisher's exact test) and (B) subjects with albinism for foveal hypoplasia grade according to the Leicester System ($p = 1$, Chi-square test for trend). For albinism, foveal hypoplasia grade 1a is marked by the absence of the extrusion of plexiform layers and presence of a nearly normal foveal pit, outer segment (OS) lengthening, and outer nuclear layer (ONL) widening; grade 1b is absence of the extrusion of plexiform layers and presence of a shallow foveal pit, OS lengthening, and ONL widening; grade 2 is absence of the extrusion of plexiform layers and foveal pit, and presence of OS lengthening and ONL widening, grade 3 is absence of the extrusion of plexiform layers, foveal pit, and OS lengthening and presence of ONL widening; grade 4 is absence of the extrusion of plexiform layers, foveal pit, OS lengthening, and ONL widening.
