# Supplementary material for: Examining Whether AOSLO-Based Foveal Cone Metrics in Achromatopsia and Albinism Are Representative of Foveal Cone Structure
Source: Transl Vis Sci Technol. 2021 May 17;10(6):22. doi: 10.1167/tvst.10.6.22 (PMC8132001; doi:10.1167/tvst.10.6.22)
Supplement: Supplement 5 [file tvst-10-6-22_s005.pdf]

Supplementary Table S1. Previous reporting of subjects with achromatopsia

**Subject ID**	Dubis et al (2014)[1]	Scoles et al (2014)[2]	Abozaid et al (2016)[3]	Langlo et al (2016)[4]	Langlo et al (2017)[5]	Georgiou et al (2019)[6]	Mastey et al (2019)[7]	Mastey et al (2019)[8]	Litts et al (2020)[9]	Georgiou et al (2020)[10]	Linderman et al (2020)[11]	Litts et al (2021)[12]
JC_0047				PCI-007	PCI-007				same ID			same ID
JC_0686											same ID	
JC_10008						same ID						
JC_10024				PCI-001	PCI-001				same ID			same ID
JC_10025				PCI-002	PCI-002							same ID
JC_10028		same ID		PCI-004	PCI-004							same ID
JC_10029				PCI-005	PCI-005							same ID
JC_10069		same ID	same ID			same ID		same ID	same ID	same ID	same ID	same ID
JC_10089		same ID		PCI-008	PCI-008		same ID		same ID			same ID
JC_10142				PCI-011	PCI-011							same ID
JC_10151				PCI-006	PCI-006				same ID		same ID	same ID
JC_10167				BPE-003	BPE-003				same ID		same ID	same ID
JC_10168				CEI-003	CEI-003							same ID
JC_10191				PCI-017	PCI-017				same ID			same ID
JC_10195				UFC-006	UFC-006				same ID			same ID
JC_10196				UFC-002	UFC-002				same ID		same ID	same ID
JC_10197				UFC-004	UFC-004				same ID			same ID
JC_10198				UFC-003	UFC-003				same ID			same ID
JC_10213				CEI-004	CEI-004							same ID
JC_10214				PCI-019								same ID
JC_10215				CEI-005	CEI-005							same ID
JC_10216				PCI-013	PCI-013				same ID			same ID
JC_10217				PCI-012	PCI-012							same ID
JC_10224				PCI-021	PCI-021				same ID		same ID	same ID
JC_10226				UFC-010							same ID	
JC_10232				CEI-001	CEI-001			same ID	same ID			same ID

JC_10247	UFC-001	UFC-001		same ID	same ID	same ID
JC_10248	CEI-008	CEI-008		same ID		same ID
JC_10249	UFC-009	UFC-009			same ID	same ID
JC_10250	UFC-008	UFC-008			same ID	same ID
JC_10256	UFC-007	UFC-007				same ID
JC_10257	UFC-005	UFC-005				same ID
JC_10258	PCI-024	PCI-024				same ID
JC_10260	CEI-006	CEI-006				same ID
JC_10300	BPE-018	BPE-018				same ID
JC_10301	BPE-012	BPE-012				
JC_10310	CEI-002	CEI-002		same ID		same ID
JC_10320	BPE-010	BPE-010				
JC_10334	CEI-011	CEI-011				same ID
JC_10335	PCI-025					same ID
JC_10409	PCI-031	PCI-031				same ID
JC_10416	CEI-009	CEI-009				same ID
JC_10417	BPE-019	BPE-019				same ID
JC_10424	PCI-020	PCI-020				same ID
JC_10490						same ID
JC_10494					same ID	
JC_10551			same ID			same ID
JC_10617						same ID
JC_10853	PCI-032			same ID	same ID	same ID
JC_10854	PCI-033			same ID	same ID	same ID
JC_10953	CEI-016					same ID
JC_10966	CEI-015					
JC_10968	PCI-034				same ID	same ID
JC_10999				same ID		same ID
JC_11000						same ID

JC_11034												same ID
JC_11036												same ID
JC_11060												same ID
JC_11061												same ID
JC_11062									same ID			same ID
JC_11066												same ID
JC_11067												same ID
JC_11091											same ID	
JC_11155												same ID
JC_11228												same ID
JC_11296												same ID
JC_11297												same ID
JC_11401												same ID
JC_11579												same ID
JC_11623											same ID	same ID
JC_11871											same ID	
JC_1208	same ID			PCI-009	PCI-009				same ID		same ID	same ID
KS_10088		same ID				same ID				same ID	same ID	
KS_10337						same ID				same ID		same ID
KS_11453												same ID
KS_11531												same ID

References

1. Dubis AM, Cooper RF, Aboshiha J, et al. Genotype-dependent variability in residual cone structure in achromatopsia: towards developing metrics for assessing cone health. *Invest Ophthalmol Vis Sci* 2014;55:7303-7311.
2. Scoles D, Sulai YN, Langlo CS, et al. In vivo imaging of human cone photoreceptor inner segments. *Invest Ophthalmol Vis Sci* 2014;55:4244-4251.
3. Abozaid MA, Langlo CS, Dubis AM, Michaelides M, Tarima S, Carroll J. Reliability and repeatability of cone density measurements in patients with congenital achromatopsia. *Adv Exp Med Biol* 2016;854:277-283.

4. Langlo CS, Patterson EJ, Higgins BP, et al. Residual foveal cone structure in *CNGB3*-associated achromatopsia. *Invest Ophthalmol Vis Sci* 2016;57:3984-3995.
5. Langlo CS, Erker LR, Parker M, et al. Repeatability and longitudinal assessment of foveal cone structure in *CNGB3*-associated achromatopsia. *Retina* 2017;37:1956-1966.
6. Georgiou M, Litts KM, Kalitzeos A, et al. Adaptive optics retinal imaging in *CNGA3*-associated achromatopsia: Retinal characterization, interocular symmetry, and intrafamilial variability. *Invest Ophthalmol Vis Sci* 2019;60:383-396.
7. Mastey RR, Gaffney M, Litts KM, et al. Assessing the interocular symmetry of foveal outer nuclear layer thickness in achromatopsia. *Transl Vis Sci Technol* 2019;8:21.
8. Mastey RR, Georgiou M, Langlo CS, et al. Characterization of retinal structure in *ATF6*-associated achromatopsia. *Invest Ophthalmol Vis Sci* 2019;60:2631-2640.
9. Litts KM, Georgiou M, Langlo CS, et al. Interocular symmetry of foveal cone topography in congenital achromatopsia. *Curr Eye Res* 2020;45:1257-1264.
10. Georgiou M, Litts KM, Singh N, et al. Intraobserver repeatability and interobserver reproducibility of foveal cone density measurements in CNGA3- and CNGB3-achromatopsia. *Transl Vis Sci Technol* 2020;9:37.
11. Linderman RE, Georgiou M, Woertz EN, et al. Preservation of the foveal avascular zone in achromatopsia despite the absence of a fully formed pit. *Invest Ophthalmol Vis Sci* 2020;61:52.
12. Litts KM, Woertz EN, Georgiou M, et al. Optical coherence tomography artifacts are associated with adaptive optics scanning light ophthalmoscopy success in achromatopsia. *Transl Vis Sci Technol* 2021;10:11.
