# Supplementary material for: Examining Whether AOSLO-Based Foveal Cone Metrics in Achromatopsia and Albinism Are Representative of Foveal Cone Structure
Source: Transl Vis Sci Technol. 2021 May 17;10(6):22. doi: 10.1167/tvst.10.6.22 (PMC8132001; doi:10.1167/tvst.10.6.22)
Supplement: Supplement 6 [file tvst-10-6-22_s006.pdf]

Supplementary Table S2. Previous reporting of subjects with albinism

**Subject ID**	McAllister et al (2010)[1]	Wilk et al (2014)[2]	Lee et al (2018)[3]	Wilk et al (2017)[4]	Wilk et al (2017)[5]	Woertz et al (2020)[6]	Woertz et al (2020)[7]
AD_11837						same ID	
AD_11897						same ID	
AD_11925						same ID	
AD_11941						same ID	
BB_10965			same ID	same ID		same ID	
DC_0831		same ID		same ID	same ID		
GS_10977				same ID			
GS_10979			same ID	same ID		same ID	
GS_11148						same ID	
GS_11807						same ID	
JC_0103	JC0103	same ID		same ID	same ID	same ID	
JC_0150		same ID		same ID			
JC_0174		same ID		same ID			
JC_0438				same ID	same ID		
JC_0456		same ID		same ID	same ID	same ID	
JC_0492		same ID	same ID	same ID	same ID	same ID	same ID
JC_0493		same ID	same ID	same ID	same ID	same ID	same ID
JC_0494		same ID					
JC_0598		same ID					
JC_0829		same ID	same ID	same ID	same ID		
JC_10042				same ID	same ID		
JC_10043				same ID			
JC_10061				same ID	same ID		
JC_10068				same ID			
JC_10073		same ID		same ID			
JC_10074		same ID		same ID	same ID		

JC_10081			same ID	same ID		
JC_10092			same ID	same ID		
JC_10093			same ID	same ID	same ID	same ID
JC_10192			same ID	same ID		
JC_10193			same ID		same ID	
JC_10227		same ID	same ID	same ID		same ID
JC_10230				same ID		same ID
JC_10269			same ID			
JC_10278		same ID	same ID	same ID	same ID	
JC_10279			same ID			
JC_10287			same ID	same ID		
JC_10496		same ID	same ID			
JC_10508		same ID	same ID		same ID	
JC_10797		same ID	same ID		same ID	
JC_10841			same ID			
JC_11046			same ID		same ID	
JC_11430		same ID			same ID	
JC_11822					same ID	
JC_11824					same ID	
JC_11849					same ID	
JC_11850					same ID	
JC_11851					same ID	
JC_11854					same ID	
JC_11899					same ID	
JC_11934					same ID	
KS_0551	same ID		same ID			
KS_0935	same ID		same ID	same ID		
KS_10314			same ID			
SS_11938					same ID	

References

1. McAllister JT, Dubis AM, Tait DM, et al. Arrested development: High-resolution imaging of foveal morphology in albinism. *Vision Res* 2010;50:810-817.
2. Wilk MA, McAllister JT, Cooper RF, et al. Relationship between foveal cone specialization and pit morphology in albinism. *Invest Ophthalmol Vis Sci* 2014;55:4186-4198.
3. Lee DJ, Woertz EN, Visotcky A, et al. The Henle fiber layer in albinism: Comparison to normal and relationship to outer nuclear layer thickness and foveal cone density. *Invest Ophthalmol Vis Sci* 2018;59:5336-5348.
4. Wilk MA, Huckenpahler AL, Collery RF, Link BA, Carroll J. The effect of retinal melanin on optical coherence tomography images. *Transl Vis Sci Technol* 2017;6:8.
5. Wilk MA, Wilk BM, Langlo CS, Cooper RF, Carroll J. Evaluating outer segment length as a surrogate measure of peak foveal cone density. *Vision Res* 2017;130:57-66.
6. Woertz EN, Omoba BS, Dunn TM, et al. Assessing ganglion cell layer topography in human albinism using optical coherence tomography. *Invest Ophthalmol Vis Sci* 2020;61:36.
7. Woertz EN, Wilk MA, Duwell EJ, Mathis JR, Carroll J, DeYoe EA. The relationship between retinal cone density and cortical magnification in human albinism. *J Vis* 2020;20:1-22.
