# Supplementary material for: Examining Whether AOSLO-Based Foveal Cone Metrics in Achromatopsia and Albinism Are Representative of Foveal Cone Structure
Source: Transl Vis Sci Technol. 2021 May 17;10(6):22. doi: 10.1167/tvst.10.6.22 (PMC8132001; doi:10.1167/tvst.10.6.22)
Supplement: Supplement 7 [file tvst-10-6-22_s007.pdf]

Supplementary Table S3. Genetic information for subjects not previously reported

Subject ID	Condition	Genotype
JC_0605	Achromatopsia	*CNGB3* c.1148delC, p.Thr383Ilefs*13
		CNGB3 c.1578+1G>A (splice site)
JC_10616	Achromatopsia	*CNGB3* c.1148delC, p.Thr383Ilefs*13
		CNGB3 c.208C>T, p.Gln70Ter
JC_10927	Achromatopsia	*CNGB3* c.1148delC, p.Thr383Ilefs*13 (hom)
		GNAT2 c.370G>A, p.Val124Met
JC_11111	Achromatopsia	*CNGA3* c.829C>T, p.Arg277Cys
		CNGA3 c.1669G>A, p.Gly557Arg
JC_11112	Achromatopsia	*CNGA3* c.848G>A, p.Arg283Gln
		CNGA3 c.1306C>T, p.Arg436Trp
JC_11486	Achromatopsia	*CNGA3* c.1641C>A, p.Phe547Leu
		CNGA3 c.955T>C, p.Cys319Arg
JC_11487	Achromatopsia	*CNGA3* c.1641C>A, p.Phe547Leu
		CNGA3 c.955T>C, p.Cys319Arg
JC_11870	Achromatopsia	*CNGB3* c.1148delC, p.Thr383Ilefs*13 (hom)
AD_11940	Albinism	*TYR* c.-4586_del4461bp_insTT
		TYR c.880G>A, p.E294K
		TYR c.1205G>A, p.R402Q
AD_11991	Albinism	*TYR* c.1205G>A, p.R402Q (hom)
		GPR143 c.-171G>A (VUS)
AD_12015	Albinism	*TYR* c.575C>A, p.S192Y
AD_12016	Albinism	*TYR* c.575C>A, p.S192Y
		TYR c.1205G>A, p.R402Q
AD_12021	Albinism	*TYR* c.575C>A, p.S192Y
		TYR c.1205G>A, p.R402Q
		OCA2 c.1327G>A, p.V443I
		HPS3 c.641C>T, p.T214M
AD_12090	Albinism	*TYR* c.1118C>A, p.T373K
		TYR c.1205G>A, p.R402Q
AD_12112	Albinism	*TYR* c.575C>A, p.S192Y
		TYR c.823G>T, p.V275F
		TYR c.1205G>A, p.R402Q
DC_10138	Albinism	*TYR* c.26T>C, p.L9P
		TYR c.980A>G, p.Y327C
		OCA2 c.1109T>C, p.I370T
DC_11476	Albinism	*TYR* c.1205G>A, p.R402Q

		BLOC1S3 c.301_302insC (hom)
DC_12122	Albinism	*OCA2* c.1025A>G, p.Y342C
		OCA2 c.1465A>G, p.N489D
GS_11902	Albinism	*TYR* c.575C>A, p.S192Y
		GPR143 c.853A>T, p.R285*
JC_10203	Albinism	*TYR* c.575C>A, p.S192Y
		TYR c.1205G>A, p.R402Q
		TYR c.1467_1468insT (fs)
JC_10228	Albinism	*TYR* c.575C>A, p.S192Y
		OCA2 c.1327G>A, p.V443I
JC_10552	Albinism	*OCA2* c.1842+2T>C (splice site)
JC_11084	Albinism	*TYR* c.1A>G, p.M1V
		TYR c.242C>T, p.P81L
		TYR c.575C>A, p.S192Y
		TYR c.1205G>A, p.R402Q
JC_11817	Albinism	*TYR* c.575C>A, p.S192Y (hom)
		TYR c.1118C>A, p.T373K (hom)
JC_11825	Albinism	*TYR* c.823G>T, p.V275F
		TYR c.1336G>A, p.G446S
JC_11842	Albinism	*TYR* c.71G>A, p.C24Y
		TYR c.230G>A, p.R77Q
JC_11895	Albinism	*TYR* c.896G>A, p.R299H (hom)
JC_11896	Albinism	*OCA2* c.1349C>T, p.T450M
		OCA2 c.1363A>G, p.R455G
		OCA2 c.1994C>T, p.A665V
JC_11898	Albinism	*TYR* c.575C>A, p.S192Y
		OCA2 c.1327G>A, p.V443I
		OCA2 c.1983G>T, p.L661F
JC_11900	Albinism	*TYR* c.575C>A, p.S192Y
		TYR c.1205G>A, p.R402Q (hom)
		OCA2 c.632C>T, p.P211L
		OCA2 c.1465A>G, p.N489D
JC_11992	Albinism	*TYR* c.575C>A, p.S192Y (hom)
		OCA2 c.1465A>G, p.N489D
		OCA2 c.2330G>A, p.C777Y
SS_11939	Albinism	*TYR* c.650G>A, p.R217Q
		TYR c.823G>T, p.V275F
		TYR c.1205G>A, p.R402Q
SS_12121	Albinism	*TYR* c.575C>A, p.S192Y (hom)

		OCA2 c.1255C>T, p.R419W
		OCA2 c.1441G>A, p.A481T
		TYRP1 c.785C>T, p.T262M
SS_12124	Albinism	*TYR* c.1205G>A, p.R402Q (hom)
		TYRP1 c.140_141delCT (fs)
		TYRP1 c.194G>A, p.C65Y
fs = frameshift, hom = homozygous, VUS = variant of unknown significance		
